# Innovations in mental health services implementation: a report on state-level data from the U.S. Evidence-Based Practices Project

**DOI:** 10.1186/1748-5908-1-13

**Published:** 2006-05-30

**Authors:** Jennifer L Magnabosco

**Affiliations:** 1 Health Science Research Specialist, VA Center for the Study of Healthcare Provider Behavior, VA Greater Los Angeles Healthcare System, Sepulveda, California, USA

## Abstract

**Background:**

The *Evidence-Based Practice *(EBP) *Project *has been investigating the implementation of evidence-based mental health practices (Assertive Community Treatment, Family Psychoeducation, Integrated Dual Diagnosis Treatment, Illness Management and Recovery, and Supported Employment) in state public mental health systems in the United States since 2001. To date, *Project *findings have yielded valuable insights into implementation strategy characteristics and effectiveness. This paper reports results of an effort to identify and classify state-level implementation activities and strategies employed across the eight states participating in the Project.

**Methods:**

Content analysis and Greenhalgh et al's (2004) definition of innovation were used to identify and classify state-level activities employed during three phases of EBP implementation: Pre-Implementation, Initial Implementation and Sustainability Planning. Activities were coded from site visit reports created from documents and notes from key informant interviews conducted during two periods, Fall 2002 – Spring 2003, and Spring 2004. Frequency counts and rank-order analyses were used to examine patterns of implementation activities and strategies employed across the three phases of implementation.

**Results:**

One hundred and six discreet implementation activities and strategies were identified as innovative and were classified into five categories: 1) state infrastructure building and commitment, 2) stakeholder relationship building and communications, 3) financing, 4) continuous quality management, and 5) service delivery practices and training. Implementation activities from different categories were employed at different phases of implementation.

**Conclusion:**

Insights into effective strategies for implementing EBPs in mental health and other health sectors require qualitative and quantitative research that seeks to: a) empirically test the effects of tools and methods used to implement EBPs, and b) establish a stronger evidence-base from which to plan, implement and sustain such efforts. This paper offers a classification scheme and list of innovative implementation activities and strategies. The classification scheme offers potential value for future studies that seek to assess the effects of various implementation processes, and helps establish widely accepted standards and criteria that can be used to assess the value of innovative activities and strategies.

## Background

During the last decade the testing and implementation of evidence-based practices (EBPs) in healthcare systems throughout the United States has increased. While published literature examining implementation of EBPs in health continues to grow [1-6], relatively few studies have focused on adult persons with serious mental illness [7-13] and fewer still on implementation processes in public mental health systems, nationally [14] or within particular states [15-19]. Relatively few authors have examined innovations in mental health, including the implementation of EBPs, within governmental systems [20-26].

The *Evidence-Based Practices (EBP) Project *[7,18] was designed to address some of these gaps. Since 2001, the *EBP Project *has been investigating the implementation of evidence-based mental health practices (Assertive Community Treatment, Family Psychoeducation, Integrated Dual Diagnosis Treatment, Illness Management and Recovery, and Supported Employment) in state public mental health systems for adult persons with serious mental illness. A key objective of the *Project *has been to collect data that help to better understand barriers and facilitators to the implementation of EBPs in mental health service delivery, as well as how stakeholders in community-based and state agencies interact to implement, achieve and sustain evidence-based service delivery cultures.

The EBP project's primary objective responds to calls for the development of a theoretical and empirical knowledge base to support the implementation and evaluation of EBPs throughout the mental health sector. Torrey et al [26] note that "The literature has an abundance of evidence, whether it is theoretical or empirical, which chronicles the arguments for the need for innovation in mental health services implementation...." Other authors have highlighted the need for research to define and identify innovations [2,27,28], particularly innovations grounded in theory and practice [28-30], as well as efforts to identify and evaluate effective innovations [28-31] and plan their widespread dissemination [3].

Progress in developing theory and empirical evidence to support implementation efforts requires the development of standards and criteria to identify, assess and utilize innovations in mental health services implementation (e.g., new administrative or clinical practices, new actions or interventions used to implement EBPs). Although systematic reviews have examined how innovations are implemented [2], and evaluations of innovations in government and organizations that partner with government have been occurring for many years [32,33], no critical reviews exist that assess the pool of evaluation criteria, methods and tools that currently are, or have been, used by government and other human service delivery organizations.

To date, only the National Science Foundation [34] has undertaken a systematic process to examine the myriad of issues (e.g., leadership is essential to innovation [30]) and questions (e.g., "How long does innovation need to run before we see effects? Does innovation fit the pattern of how government works?" [27]) that have been raised by the innovations literature and other sources in the quest to develop widely accepted standards and criteria. Hence, policy and practice leaders, researchers, and other stakeholders in the mental health and healthcare fields lack a strong evidence-base from which to select appropriate tools, activities and strategies that might help produce more effective mental health [12] and healthcare services for vulnerable populations, such as persons with serious mental illness.

The research reported in this paper was designed to generate such evidence by examining the range activities and strategies employed to implement the *Project's *EBPs in public mental health systems. It addressed the following research questions:

• Can innovative implementation activities be identified from data sources that describe the processes, activities and methods states used to prepare, initially implement and plan for sustainability of the *EBP Project*?

• What types of innovative implementation activities were employed during the three phases of implementation?

This paper describes a framework for identifying and classifying the activities and strategies state mental health (and substance abuse) authorities employed during Pre-Implementation, Initial Implementation, and Sustainability Planning phases of the *EBP Project*.

## Methods

This study was a secondary analysis of site visit reports documenting state-level activities and strategies associated with the implementation of five EBPs in eight states during the Pre-Implementation, Initial Implementation, and Sustainability Planning phases of the *EBP Project *during two observational periods.

A complete description of the *Project's *EBPs, state and community-based site selection, EBP training materials, and agency site level implementation and evaluation methods can be found in Torrey et al [6] and on the websites for the Dartmouth Psychiatric Research Center [35] and the Evidence Based Practices Project Phase I Steering Committee [36].

### Setting and participants

The eight states selected for participation in the initial round of the *EBP Project *were recruited at national meetings and through *Project *announcements. A process for state and EBP selection was developed and approved by *Project *developers, researchers and funding agencies.

Researchers associated with the John D. and Catherine T. MacArthur Foundation Network on Mental Health Policy Research-and the National Association of State Mental Health Directors Research Institute (NASMHPD NRI) – identified the types of key informants for individual and group interviews suitable for the study's interview protocol topics (see below), and also sought to gain expert and multiple perspectives on state EBP activities and strategies. Key informants in each state included: state mental health and state substance abuse agency directors; state EBP *Project *implementation team members; outpatient mental health and substance abuse community-based *Project *site agency directors; state managers of finance, Medicaid, research, quality assurance, training, vocational rehabilitation, and supported employment; representatives of consumer groups, such as National Alliance for the Mentally Ill (NAMI) or the state's Consumer Affairs Office representative; consumers from *Project *sites; and consultants with whom states or agencies contracted to assist in the implementation of the *Project *EBPs.

### Data collection

Network and NRI researchers conducted site visits in each of the participating states at state department of mental health offices during a 1-2 day period during each observational period. Time 1 site visits were conducted during Fall 2002 and Spring 2003 to investigate state-level activities and strategies associated with the Pre-Implementation and Initial Implementation phases of the *Project*. Follow-up site visits at Time 2 were conducted during Spring 2004 to investigate the continued Initial Implementation and Sustainability Planning phases of the *Project*.

Key informant interview protocols for the two observational periods were developed by the Network and NRI researchers in consultation with various experts in the field of mental health, including consumers. Protocols were informed by research in fields such as diffusion of innovations, implementation, organizational theory, EBP and healthcare delivery. More than 50 interviews were conducted in all states, with 1-2 individual and 5-8 group interviews conducted in each of the eight states during each time period.

The Time 1 interview protocols included questions about the organization of state mental health systems, the state role in EBP implementation, and EBP characteristics, including the status of implementation, monitoring and feedback mechanisms, and initial plans for state-wide implementation and sustainability. Analysis of Time 1 site visit reports revealed three main areas of interest that were used to focus inquiry during Time 2 site visits: leadership and political environments associated with state mental health systems, financing and regulations associated with EBP implementation, and quality and training associated with EBP implementation, measurement and use of outcome data. Time 2 protocols included questions highlighting these three main areas and also similar questions from Time 1, so that continuity and progress of activities and strategies could be assessed.

Sixteen site visit reports were written by designated research team members who conducted the site visit interviews and/or served as note takers. Initial drafts of reports were approved by all team members and were sent to the state *EBP Project *team for review and validation. Revisions to reports were made as needed and considered valid after states and research team members approved final versions.

Site visit reports synthesized data collected on each state's activities and strategies using a profile report format developed for the *Project*. Data included: site visit key informant interview notes for individual and group interviews, research team site visit debriefing meeting notes, background information collected on states (i.e,, reports and other documents describing state systems and EBP activities), and annual state profile data posted on NASMHPD's website, . The sixteen site visit reports were the data sources used in the current study.

### Data coding and analysis

Content analysis techniques [37] were employed to identify, code and categorize the state-level activities and strategies associated with three stages of diffusion or implementation described by Rogers [38] and Greenhalgh et al [2]: Pre-Implementation or "readiness" for implementation (Time 1 of this study), Initial Implementation (active and planned efforts to mainstream an innovation, or EBP, within organizations; Times 1 and 2 of this study), and Sustainability Planning for the EBPs (Time 2 of this study).

Implementation activities and strategies were considered innovative if they were specifically intended to launch, implement and/or enhance the implementation of the *Project's *EBPs, according to Greenhalgh et al's definition for innovation [2] – "a novel set of behaviours, routines, and ways of working that are directed at improving outcomes, administrative efficiency, cost effectiveness, or users' experiences that are implemented by planned and coordinated actions." Implementation activities and strategies were considered "novel" if they were newly developed to prepare for, initially implement or plan for sustainability of the *Project's *EBPs. For example, "partnerships to train stakeholders" (see Table 3) were coded as innovative when they were newly established for implementation of a *Project *EBP. Training partnerships were not considered innovative if they existed prior to the launch of the *Project *and modified an existing training module to implement a *Project *EBP.

**Table 3:** Pre-Implementation Phase: Innovative Implementation Activities and Strategies for Project EBPs*

**Innovations: Pre-Implementation**	**ACT**	**FPE**	**IDDT**	**IMR**	**SE**
***State Infrastructure Building and Commitment***					
• Technical Assistance Center for state and Toolkit efforts established	X				
• Participation in other demonstrations to ready state for EBPs					X
• Modifications to Toolkit made to fit state context of implementing EBPS	X			X	
• White Paper written by consumers to modify EBP				X	
• State sponsored research establishing evidence base to implement EBPs			X	X	
***Stakeholder Relationship Building and Communication***					
• State-wide meetings, workshops, conferences, technical assistance activities to address philosophical and clinical practice differences between providers			X	X	
• Broad communication strategies established (e.g. educational forums, peer support programs, statewide consumer and advocacy meetings) to discuss EBPs		X		X	
• State-wide meetings to engage consumers and other stakeholders in state and Toolkit efforts	X	X	X	X	
• State-wide Advisory Group established	X	X	X	X	
• State-wide Advisory Committee established, integrating recovery perspectives				X	
• Priority to include input and consumers on Advisory Board, Toolkit site Steering Committees	X	X	X	X	X
• Reporting of current EBP successes in mass media					X
• Partnership formed between state and consumer community to train clinical staff		X			
***Financing***					
• Start-up incentive monies for sites provided by state	X	X	X	X	X
• Start-up incentive monies for sites provided by non-state funder				X	X
• New use of block grant funds to support EBPs		X			X
• Shift of funding from inpatient to community services by state	X				
• Financial incentives, using Medicaid billing, for start-up year	X				
• Approaches to make Medicaid billing easier for EBPs investigated by state	X	X	X	X	X
• Education and assurance about Medicaid billing procedures provided to sites by state					X
• White paper written by consumers to address Medicaid reimbursement and coding issues				X	
• MOUs signed by community mental health centers to receive start-up funds		X			
• State Vocational Rehab Agency established MOUs to solidify payment for services					X
***Continuous Quality Management***					
• New licensing standards developed by non-state experts	X				
• New licensing regulations developed or discussed	X	X			X
• New dual certification and licensing standards established			X		
• New standards for service delivery established	X				X
• Association for Behavioral Health Centers formed to discuss reimbursement and administrative rules and incentives for clinical staff to perform services		X			
***Service Delivery Practices and Training***					
• Training budget reallocated to be more effective for EBPS		X			X
• Two-year training plan developed through community needs assessment process to deliver training through regional training centers					X
• Tracks in clinical supervision and clinical administration best practices developed by state			X		
• Sites to receive incentives for additional training and technical assistance if decide to implement EBP			X		

* EBPs:

ACT = Assertive Community Treatment

FPE = Family Psychoeducation

IDDT = Integrated Dual Diagnosis Treatment

IMR = Illness Management and Recovery

SE = Supported Employment

An inductive analysis approach [38], allowing patterns, themes and categories to emerge from the data, was used to classify the activities and strategies identified. Therefore, categories of activities and strategies were evaluator generated [38]. Since the number of states involved in the study was small, and full case studies of the states were not conducted, analyses focused on themes or common categories of innovative implementation activities across the EBPs implemented, and trends in the use of these activities. Analyses included the determination of the rank order of innovative implementation activities, per category, for each implementation phase (highest rank was assigned to categories with the greatest number of innovative implementation activities or strategies), as well as identification and counts of state implementation activities (e.g., mental health system reforms and other improvements in service delivery), challenges and other factors (e.g. budget crises) that provided broad-based contexts for implementing EBPs.

## Results

Table 1 shows the distribution of EBP selection by the states. States selected specific EBPs for various reasons, including: a) compatibility with established state mental health, substance abuse, or vocational rehabilitation goals, b) similarity to service practices already implemented, and/or c) to expand beyond current services by implementing new service practices for targeted populations.

**Table 1:** State Selection of EBPs *

EBP: STATE:	ACT	FPE	IDDT	IMR	SE	TOTAL
1	X		X			2
2	X			X		2
3		X			X	2
4			X		X	2
5					X	1
6		X		X		2
7		X		X		2
8			X	X		2

Total	2	3	3	4	3	15

* EBPs:

ACT = Assertive Community Treatment

FPE = Family Psychoeducation

IDDT = Integrated Dual Diagnosis Treatment

IMR = Illness Management and Recovery

SE = Supported Employment

Table 2 shows the number of innovative implementation activities by category and implementation phase. A total of 106 discreet innovative implementation activities and strategies were identified. Content analysis produced five broad categories of activities and strategies:

**Table 2:** Number and Category of State-Level Implementation Activities and Strategies across Implementation Phases

CATEGORY	PRE-IMPLEMENTATION	INITIAL IMPLEMENTATION	SUSTAINABILITY PLANNING	TOTAL
State Infrastructure Building and Commitment	5	3	11	19
Stakeholder Relationship Building and Communication	8	9	6	27
Financing	5	13	12	26
Continuous Quality Management	5	9	3	17
Service Delivery Practices and Training	4	5	8	17

Total	27	39	40	106

• State infrastructure building and commitment;

• Stakeholder relationship building and communication,

• Financing;

• Continuous quality management, and

• Service delivery practices and training.

Tables Table 3, Table 4, Table 5 contain a list of activities associated with each implementation phase, category and EBP. The remainder of this section describes activities and strategies by implementation phase with the most prevalent category discussed first.

**Table 4:** Initial Implementation Phase: Innovative Implementation Activities and Strategies for Project EBPs*

**Innovations: Initial Implementation**	**ACT**	**FPE**	**IDDT**	**IMR**	**SE**
***State Infrastructure Building and Commitment***					
• New state position developed to assist in implementation and monitoring of EBPs established			X	X	
• SMHA considering strategies to penetrate EBP in all licensed programs				X	
• New RFP process developed to help fund EBP projects throughout state					X
***Stakeholder Relationship Building and Communication***					
• Monthly meetings between state, Toolkit sites, and/or Advisory Councils	X	X	X	X	X
• Monthly meetings between NAMI and Toolkit sites				X	
• Monthly meetings and/or calls between technical assistance centers and sites	X		X	X	
• Ongoing communication between state and local sites/boards			X	X	
• Increased collaboration between SMHA and State Medicaid Office	X	X	X	X	X
• New collaboration between SMHA, Medicaid and Vocational Rehab Office					X
• First time meeting held between state NAMI and Office of Consumer Affairs directors			X		
• State and local sites working to implement evaluation process and reassure stakeholders of process		X			
• Developed Clinical Practices Advisory Committee				X	
• Planning EBP conference	X				X
***Financing***					
• SMHA working with State Medicaid agency to make billing easier			X		
• Developed new Medicaid billing code and coding guidelines		X	X		
• Using bundled funding approach to fund EBP	X				
• Exploring Medicaid requirements to qualify consumers to deliver EBP		X			
• Using Medicaid Waiver 1115B to fund EBP		X			
• Position paper written by state to recommend Medicaid reimbursement levels and codes				X	
• Billing of EBP allowed as part of group or individual psychotherapy or day rate for Continuing Day Treatment Program				X	
• Reimbursement codes and rates changed to support EBP					X
• Created new funding program only for EBP					X
• New funding formulas integrated into allocation structure, with codes changed in data system and audit process	X				
• Medicaid approval received to reimburse EBP teams through amendment to state plan	X				
• Medicaid rate recalculated to allow more professionals to be reimbursed	X				
• State cost sharing with counties to fund EBPs	X			X	
***Continuous Quality Management***					
• Distributed SAMSHA's standards of care to local sites			X		
• Developed and using new certification manual	X				
• Developing treatment plan tool to include multiple domains and to be consistent with licensure review			X		
• Developing mental health and substance abuse language guidelines for auditors to use in consistent evaluations			X		
• Developing standards for EBP					X
• Barriers to standards for EBP teams removed by Medicaid agency	X				X
• Regulation changes to revise employment referral and authorization form, individual vocational form and verification of diagnostic process, and employment outcome measurement definition					X
• Implementing certification process through administrative rule and stakeholder process	X				
• Integrated fidelity measures, technical support and supervision into certification	X				
***Service Delivery Practices and Training***					
• Developing treatment plan tool to include multiple domains and to be consistent with licensure review			X		
• SMHA and consumer community developing partnership to train clinical staff to deliver EBP				X	
• SMHA funding for consumer training and joint teaching to professionals and consumers for EBP				X	
• Implementing shadowing training program	X				X
• Administrative rule revised to include fidelity adherence for EBP					X

* EBPs:

ACT = Assertive Community Treatment

FPE = Family Psychoeducation

IDDT = Integrated Dual Diagnosis Treatment

IMR = Illness Management and Recovery

SE = Supported Employment

**Table 5:** Sustainability Planning Phase: Innovative Implementation Activities and Strategies for Project EBPs*

**Innovations: Sustainability Planning**	**ACT**	**FPE**	**IDDT**	**IMR**	**SE**
***State Infrastructure Building and Commitment***					
• Commitment to state-wide rollout no matter resources needed	X	X	X	X	X
• State and sites committed to rollout of EBP together			X		
• Goal assess fidelity before rolling out EBP		X		X	
• Goal to re-examine EBP and retrofit rollout because of nature of EBP	X		X	X	
• Goal to examine difference between EBP rollouts because of difference between EBPs and paradigm shifts required to implement			X		X
• Goal to determine system-level adaptations perceived to be required for sustained uptake			X		
• State applying for governmental grants to build system infrastructure	X				
• Plan to implement a state institute to support EBPs	X		X	X	X
• Issues for systematic implementation of EBP identified					X
• Develop infrastructure and mechanisms for integrating EBPs into larger state agenda and dissemination of EBP information across states	X	X	X	X	X
• To continue state supported research on EBPs			X	X	
***Stakeholder Relationship Building and Communication***					
• Need to develop engagement process to involve non-Toolkit agencies in EBPS more	X	X	X	X	X
• Increase family involvement in planning and monitoring community based programs		X			
• Continue to create champions at all levels of system		X			
• Continue regular consumer and stakeholder meetings	X	X	X	X	X
• Continued guidance on consensus building	X	X	X	X	X
• Develop language about EBPs that consumers can better understand and use			X	X	
***Financing***					
• Need to better align incentives and rules to encourage desired practices, behaviors and system change	X	X	X	X	X
• To work on funding base for full roll out	X		X	X	X
• To explore regulating EBPs			X		
• To develop new contract language for EBPS using administrative rule			X		
• To explore developing private insurance program to pay for EBP			X		
• To explore increasing tax on alcohol and tobacco to fund EBP			X		
• To explore expanding ACT to share financing with other EBPs			X		
• To consider higher reimbursement rates			X		X
• To explore restructuring Medicaid plan to cover services			X		
• To add EBP to Medicaid Rehab Option			X		
• To explore solid payment mechanisms		X			
• Determine how to shorten timeframes to transfer funds from the state to sites					X
***Continuous Quality Management ***					
• To work on credentialing and licensing issues with locals			X		
• Considering strategies to penetrate EBP in all licensed programs				X	
• Considering deeming EBP training part of certification process			X	X	
***Service Delivery Practices and Training***					
• State working with Schools of Social Work to develop EBP training curriculum for students	X			X	
• State to use private donation to create educative training center for EBPs	X		X		
• To address ongoing skills training	X	X	X	X	X
• To explore appropriate outcome measurement of EBP		X		X	
• To implement Train the Trainer Program	X		X	X	
• State to set aside monies for training activities	X				
• To explore strategies that achieve broader penetration of training and use of learning collaboratives	X				
• To increase access to transportation to receive EBP		X			

* EBPs:

ACT = Assertive Community Treatment

FPE = Family Psychoeducation

IDDT = Integrated Dual Diagnosis Treatment

IMR = Illness Management and Recovery

SE = Supported Employment

### Pre-Implementation phase

Rank-order and frequency analyses revealed several patterns of usage of the implementation strategies. In the Pre-Implementation phase, *stakeholder relationship building and communication *activities were most prevalent. This phase involved foundation building or macro-level activities (processes by which higher level management in government executes its influence on lower level managers and workers who implement policies, programs and laws [39] to prepare for the initial implementation of the EBPs. States employed one main relationship building and communications activity across all EBPs, prioritizing the participation of consumers on Project Advisory Boards and *EBP Project *Steering Committees.

State infrastructure and financing innovations were employed for all EBPs, except Family Psychoeducation, during the Pre-Implementation (and Initial Implementation) phases. Limited use of these activities for Family Psychoeducation was largely due to the fact that this EBP required more intensive stakeholder consensus building to incorporate its newness into practice. New licensing regulations were under development or discussion for all EBPs. State training budgets were reallocated to provide more training for the Family Psychoeducation and Supported Employment EBPs especially.

### Initial Implementation phase

During the Initial Implementation phase, *financing *was most prevalent. Initial Implementation involved a focus on resources, including financial activities and strategies to support the implementation process (e.g., organizational change, training, and monitoring efforts) of EBPs at the community-based agency level. In this phase, much attention was paid to developing strategies to fund, and develop and implement effective billing procedures for Assertive Community Treatment.

*Stakeholder relationship building and communication *activities in this phase included monthly meetings between representatives from the states and *EBP Project *sites and/or Advisory Councils for all EPBs. Additional activities in this category during this phase included increased collaboration between the state mental health and Medicaid agencies to make billing easier. While *continuous quality management *activities were most prevalent for Assertive Community Treatment in this phase, some attention to these issues was associated with all EBPs. Within this category, a shadowing training program for Assertive Community Treatment and Supported Employment was among the novel service delivery and training activities. Relatively few activities occurred in the *state infrastructure building and commitment *category in this phase, although several significant activities were employed for the Integrated Dual Diagnosis Treatment, Illness Management and Recovery, and Supported Employment EBPs. For example, one state developed a new state-level position to assist in the implementation and monitoring of the Integrated Dual Diagnosis Treatment and Illness Management and Recovery EBPs. Another state was considering strategies to penetrate Illness Management and Recovery in all licensed programs, while another developed a new RFP process to help fund the Supported Employment EBP state-wide.

### Sustainability Planning phase

As during the Initial Implementation phase, *financing *activities were most prevalent in the Sustainability Planning phase. Overall state commitment to EBP rollouts focused on intent to do so and/or targeted infrastructure building for EBPs. In this phase, states projected that they would need to prioritize securing resources – money and staff – to sustain the *Project's *EBPs after the *Project *ended. Despite serious state budget crises occurring during the time of the site visits, states expressed a philosophical commitment to rolling out all EBPs, no matter the resources needed. States were committed to developing a funding base for roll-out of all EBPs except Family Psychoeducation, as they wanted to better assess this EBP's fidelity and potential funding mechanisms. With regard to particular EBPs, planning for Integrated Dual Diagnosis Treatment was most prevalent in this phase, as it required much effort to find common philosophical ground and integrate efforts between mental health and substance abuse providers. However, states planned to better align incentives and rules to encourage desired practices, behaviors, and system change for all of the EBPs.

States also had plans to: disseminate EBP information state-wide for all of the EBPs; further develop their infrastructure and mechanisms for integrating EBPs into the larger state agenda; apply for governmental grants to build system infrastructure for Integrated Dual Diagnosis Treatment; implement a state institute to support *Project *and non-*Project *EBPs; continue state supported research for EBPs, especially for Integrated Dual Diagnosis Treatment and Illness Management and Recovery; and address the ongoing skills training, including certification and licensing needed for all EBP service delivery, especially for Integrated Dual Diagnosis Treatment and Illness Management and Recovery. State plans for sustaining *Project *EBPs through *stakeholder relationship building and communication *activities were largely built on activities set into motion during the Pre-Implementation and Initial Implementation phases.

## Discussion

This study employed qualitative data analysis methods to identify and classify 106 innovative state-level implementation activities and strategies into five distinct categories. The classification scheme and list of activities and strategies offer a framework for categorizing and studying the spectrum of activities and strategies associated with implementation of mental health EBPs at state and community levels.

This study has several limitations. Because the original data collection occurred during two cross-sections of time, and during the initial implementation of the *Project's *EBPs, it was not possible to assess the full range of implementation activities and strategies employed throughout the *Project*. In addition, the activities were identified from secondary sources, and only activities coded as innovative were included. As a result, the 106 implementation activities represent a subset of the full range of activities and strategies employed to implement the *Project's *EBPs. Other activities and strategies might be identified through longitudinal and/or more in-depth case study data collection methods. In addition, the implementation activity coding was performed by a single researcher without replication. Identification and classification decisions reached by the author might differ from those reached by other researchers.

Regardless of its limitations, this study provides new evidence that EBPs in state mental health systems are being implemented within an "evolutionary" framework [40]. Efforts to assess innovations in mental health services implementation have been hampered by the limited body of evidence regarding the validity of four classic models of implementation – evolutionary [40], adaptive [41], top down or "forward mapping" [42], and bottom up or "backward mapping" [43]. The evolutionary model addresses the shortcomings of the top-down, bottom-up, and adaptive approaches [44], recognizing that implementation-related interactions occur on various levels in multiple directions, such as between actors at different levels within an organization and across policy and practice domains [44,45].

Evolutionary implementation is considered a "continuum in which an interactive and negotiative process [takes] place over time, between those seeking to put policy [or practice] into effect and those upon whom action depends" [44]. Implementation generally occurs through "progressive movements" [45], "evolving" during the process itself. It takes into account a combination of micro- and macro- implementation processes, and recognizes that the institutional settings in which a policy or program is implemented can interact with and impact outcomes [39,46].

Here, activities across all implementation phases, and EBPs, were built on activities set in motion in earlier phases. For example, all states considered consumers key to mental health system reform. This philosophy laid the foundation for regular meetings, and Advisory Groups and technical assistance activities to take place between the state agencies, consumers and other stakeholders. Similarly, the development and implementation of effective financing, and licensing and certification strategies followed successful completion of negotiations (involving state agencies, service delivery organizations, consumer and other stakeholders) to develop new billing codes, incentives, funding streams, regulations and standards.

The innovations identified in this study show that "interactions...occur [ed] on various levels, between top and bottom actors" – and that a variety of "interrelationships" [44,45] were necessary to launch, initially implement, and plan for the sustainability of the *Project *EBPs. Here, state agencies exercised their authority to set policy for the delivery of clinical practice, and voluntarily engaged in an interactive and cooperative relationship, building process with local service and other organizations to meet the full range of needs necessary to solidify EBPs as usual mental health and administrative practice. Therefore, interactions between the macro- or top down actors (state agencies) and the micro- or bottom-up actors (local service organizations or boards) were required to successfully implement and plan for the roll-out of the EBPs.

Lastly, the variety of 'institutions" represented in this study – including but not limited to state agencies of mental health, substance abuse, Medicaid, and vocational rehabilitation, as well as universities, community-based organizations, consumer organizations, local and accrediting boards, and research groups – engaged in a variety of inter-relationships to implement the *Project's *EBPs. Consequently, "institutions matter [ed]" [47] in this study.

## Conclusion

Insights into effective strategies for implementing EBPs in mental health and other health sectors require qualitative and quantitative research that seeks to: a) empirically test the effects of tools and methods used to implement EBPs, and b) establish a stronger evidence-base from which to plan, implement, evaluate and sustain such efforts. This paper offers a classification scheme and list of implementation activities and strategies employed by eight states participating in the *EBP Project *during its initial implementation. The classification scheme offers potential value for future studies that seek to assess the effects of various implementation processes, and helps establish widely accepted standards and criteria that can be used to assess the value of innovative activities and strategies.

States employed a diverse range of implementation activities and strategies to address barriers to implementing EBPs [23,26,48] within various social, economic and political contexts [21,22,46,49-52]. These data help to continue to build evidence that the state's role is significant to the implementation of mental health service and system reform efforts [21,22,53-56]. This study also highlights the potential value of one theoretical framework – the evolutionary model of implementation – in improving understanding of the processes occurring with EBP implementation efforts.

## Competing interests

The author(s) declares that she has no competing interests.
